# Auditory and speech outcomes of cochlear implantation in patients with Waardenburg syndrome: a meta-analysis

**DOI:** 10.3389/fneur.2024.1372736

**Published:** 2024-07-11

**Authors:** Feng Qin, Siquan Guo, Xiuwen Yin, Xiaoyu Lu, Jing Ma

**Affiliations:** ^1^The Changzhou Clinical College of Xuzhou Medical University, Changzhou, Jiangsu, China; ^2^Department of Otorhinolaryngology, Changzhou Third People’s Hospital, Changzhou, Jiangsu, China; ^3^Department of Otorhinolaryngology, The First People’s Hospital of Changzhou, Changzhou, Jiangsu, China

**Keywords:** Waardenburg syndrome, cochlear implantation, auditory, speech, meta-analysis

## Abstract

**Objective:**

This study aims to assess the potential efficacy of cochlear implantation as a treatment for patients with Waardenburg syndrome (WS) and to guide clinical work by comparing the effect of auditory and speech recovery after cochlear implantation in patients with WS and non-WS.

**Methods:**

PubMed, the Cochrane Library, CNKI, and Wanfang Data were sources for retrieving literature on cochlear implantation in WS, and clinical data meeting the inclusion criteria were meta-analyzed using RevMan5.41.

**Results:**

A total of nine articles were included in this study, including 132 patients with WS and 815 patients in the control group. Meta-analysis showed that there are no significant differences in the scores for categories of audit performance (CAP), speech intelligibility rating (SIR), and parents’ evaluation of aural/oral performance of children (PEACH) between the WS group and the control group.

**Conclusion:**

Cochlear implantation demonstrates comparable auditory and speech recovery outcomes for WS patients and non-WS patients.

## 1 Introduction

Waardenburg syndrome (WS), discovered and named by Dutch physician Waardenburg in 1951 (1), is an autosomal dominant genetic disorder primarily characterized by auditory pigmentary abnormalities. Its key manifestations include inner canthus heterotopia, iris heterochrony, white hair on the forehead, and hereditary sensorineural deafness (2). WS is closely related to the abnormal migration and differentiation of melanocytes. In the inner ear, melanocytes differentiate into intermediate cells within the stria vascularis of the cochlea (3, 4). When gene mutations affect melanocyte differentiation and migration, they may influence the cochlea’s internal environment, resulting in sensorineural hearing loss.

Cochlear implantation (CI) stands out as the primary approach for auditory and speech therapy (5). Currently, there exists a scarcity of research samples and variations in evaluation criteria for assessing the effectiveness of CI in WS patients. Therefore, this paper aims to conduct a comprehensive literature review, identify common evaluation indicators, and evaluate the therapeutic impact of CI on patients with WS.

## 2 Materials and methods

This meta-analysis was performed in line with the preferred reporting items for systematic reviews and meta-analyses (PRISMA) guidelines (6). This review was also registered on PROSPERO (Registration ID: CRD42022356957).

### 2.1 Literature search

A comprehensive search strategy was implemented by combining subject words with free words across multiple databases, including PubMed, the Cochrane Library, CNKI, and Wanfang. Both English and Chinese languages were utilized for the search. The exploration period extended from the establishment time of each database to December 31, 2023. The keywords employed in the search encompassed “Waardenburg syndrome,” “cochlear,” “cochlear implant,” and “cochlear implantation.”

### 2.2 Literature inclusion and exclusion criteria

#### 2.2.1 Literature inclusion criteria

1. The inclusion criteria encompass randomized controlled trials (RCTs), cohort studies, case–control studies, or comparative studies.
2. The study population should consist of individuals undergoing auditory rehabilitation through CI.
3. The evaluation should focus on the assessment of auditory and/or speech skills in patients who have undergone CI.

#### 2.2.2 Literature exclusion criteria

1. Articles lacking comparable auditory and speech outcomes between WS and other CI patients.
2. Studies that did not specifically explore auditory rehabilitation through CI.
3. Articles with a high risk of bias.

### 2.3 Data collection and extraction

Three authors independently conducted data extraction from the full texts of eligible articles. The following data were recorded: the first author’s name, publication year, the number of patients enrolled in each study, postoperative evaluation indicators, and recovery rates. Discrepancies were resolved through discussions among the authors.

### 2.4 Quality assessment

The risk of bias was assessed using the Newcastle Ottawa Scale (NOS). Two evaluators conducted independent assessments of the literature, and a final evaluation was performed by a third party. This third party, a senior chief physician, possessed extensive clinical research experience. A score of ≥6 is considered high-quality literature, and < 6 is not included.

### 2.5 Statistical methods

RevMan5.41 software was used for the analysis. A Q-test was used for the heterogeneity test. If the heterogeneity is low (*p* > 0.1, *I*^2^ < 50%), the fixed-effect model was selected; if the heterogeneity is high (*p* ≤ 0.1, *I*^2^ ≥ 50%), the random effect model was used to carry out sensitivity analysis on the source of heterogeneity.

## 3 Results

### 3.1 Literature search results

**Figure 1:**
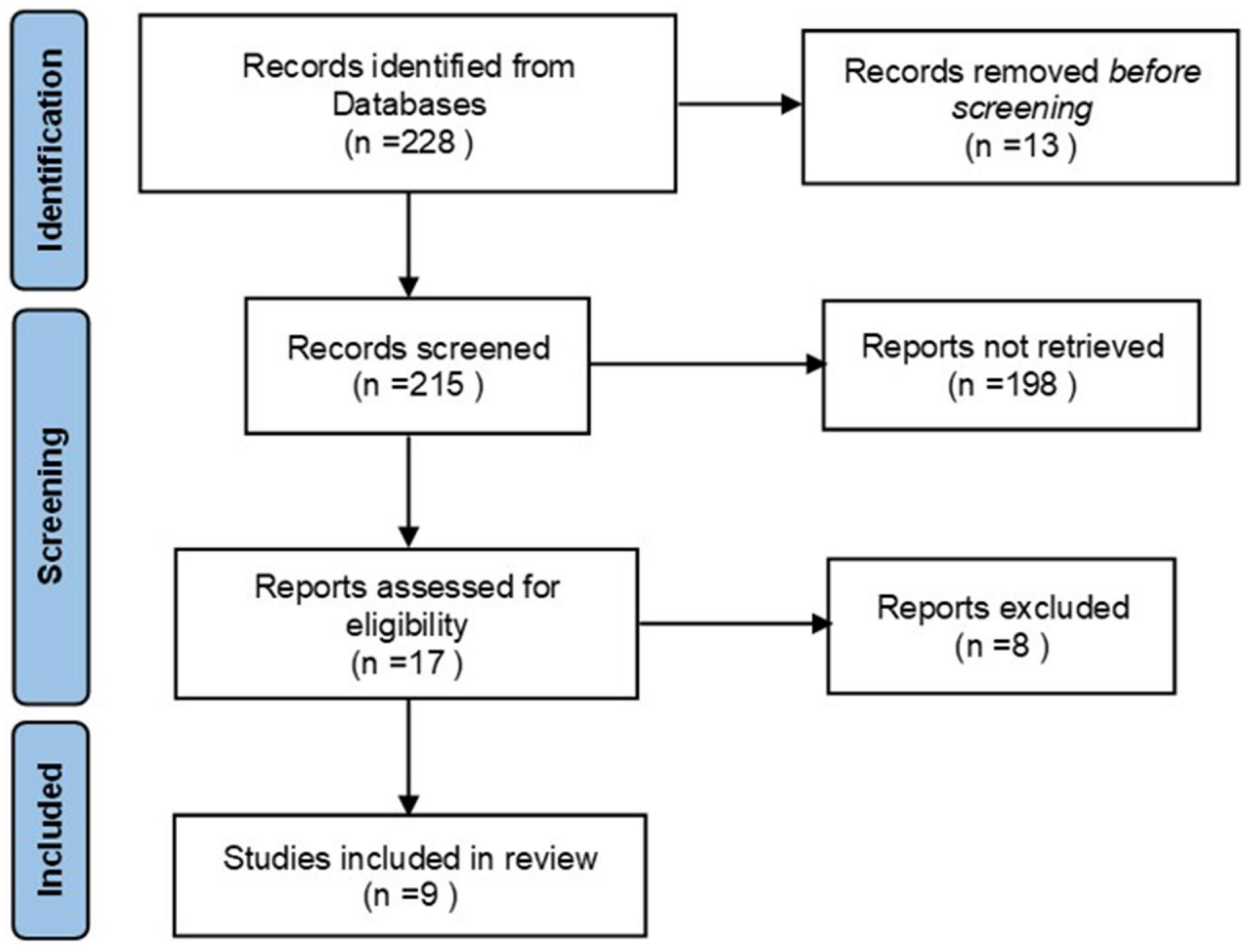
Process of literature screening.

A total of 228 documents were retrieved, finally, nine qualified documents were selected for analysis (7–15), as shown in Table 1, The literature screening process is shown in Figure 1.

**Table 1:** The basic features of the included study.

Objects	Groups	Cases	Implanted age	Male:female	Outcomes	NOS
Amirsalari 2012	WS	6	26.00 ± 15.78 months	3:3	CAP, SIR	6
	Control	75	54.48 ± 14.76 months	35:40		
Andrade 2012	WS	7	30.6 ± 9.7 months	4:3	CAP, SIR, MAIS, MUSS	8
	Control	261	36.7 ± 18.6 months	148:113		
Bakkouri 2012	WS	30	4.8 ± 3.5 years	-	CSW-OSW	6
	Control	85	4.7 ± 3.4 years			
Chu 2017	WS	8	3.52 years	5:3	CAP, SIR	7
	Control	30	3.49 years	18:12		
Dong 2013	WS	21	4.2 years	11:10	CAP, SIR, PTA, PEACH	7
	Control	21	4.3 years	11:10		
Gao 2018	WS	6	-	-	CAP, SIR	6
	Control	233				
Nierop 2016	WS	14	1.61 years	6:8	Phoneme score, RDLS LQ	8
	Control	48	1.32 years	24:24		
Zhang 2009	WS	16	4 years	9:7	PEACH	7
	Control	32	4 years	19:13		
Zhang 2022	WS	24	2.29 ± 0.78 years	16:8	CAP, SIR	8
	Control	30	2.35 ± 0.98 years	18:12		

NOS, Newcastle Ottawa Scale; CAP, Categories of audit performance; SIR, Speech intelligibility rating; MAIS, Meaningful auditory integration scales; MUSS, Meaningful use of speech scale; CSW-OSW, Closed-set and open-set words; PTA, The pure tone audiometry; PEACH, Parents’ evaluation of aural/oral performance of children; RDLS, The Reynell Develop-mental Language Scales; LQ, Language quotient.

### 3.2 Literature heterogeneity test

Heterogeneity tests were conducted for each test, and fixed-effects model analysis was performed on categories of audit performance (CAP), speech intelligibility rating (SIR), and parents’ evaluation of aural/oral performance of children (PEACH) scores (*p* > 0.05, *I*^2^ < 50%).

### 3.3 Comparison of postoperative CAP scores

**Figure 2:**
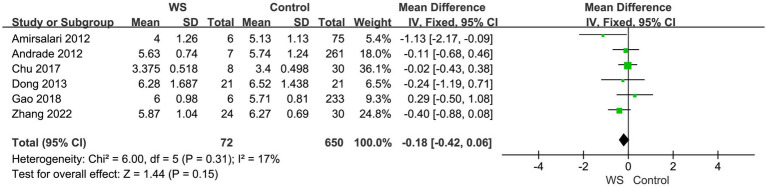
Forest map of CAP comparison between the WS group and the control group.

Six studies, involving a total of 722 cases (72 cases in the WS group and 650 cases in the control group), compared the CAP scores of the WS group and the control group after CI. The fixed-effect model was used for analysis, and the combined-effect test result yielded *Z* = 1.44, *p* = 0.15. This suggests that there is no statistical difference between the WS group and the control group in terms of CAP scores, as shown in Figure 2.

### 3.4 Comparison of postoperative SIR scores

**Figure 3:**
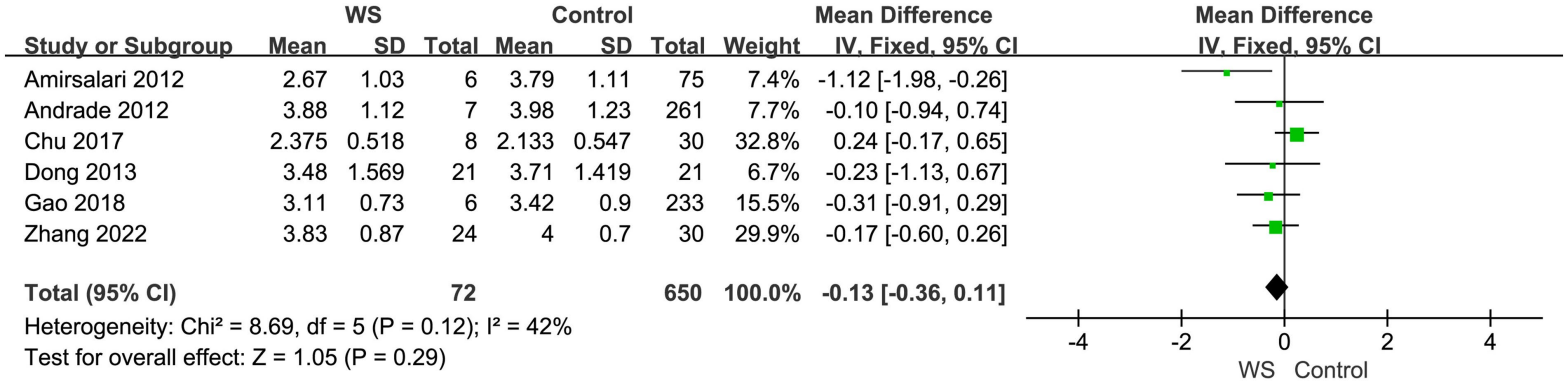
Forest map of SIR comparison between the WS group and the control group.

Six studies, involving a total of 722 cases (72 cases in the WS group and 650 cases in the control group), compared the SIR scores of the WS group and the control group after CI. The fixed-effect model was used for analysis, and the combined-effect test result yielded *Z* = 1.05, *p* = 0.29. This indicates that there is no statistical difference between the WS group and the control group in terms of SIR scores, as shown in Figure 3.

### 3.5 Comparison of postoperative PEACH scores

#### 3.5.1 Comparison of telephone scores

**Figure 4:**
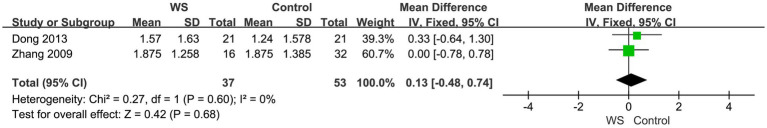
Forest map of telephone communication score comparison between the WS group and the control group.

Two studies, involving a total of 90 cases (37 cases in the WS group and 53 cases in the control group), compared the telephone scores of the WS group and the control group after CI. The fixed-effect model was used for analysis, and the combined-effect scale test result yielded *Z* = 0.42, *p* = 0.68. This reveals that there is no statistical difference between the WS group and the control group in terms of the comparison of telephone scores, as shown in Figure 4.

#### 3.5.2 Comparison of quiet environment scores

**Figure 5:**
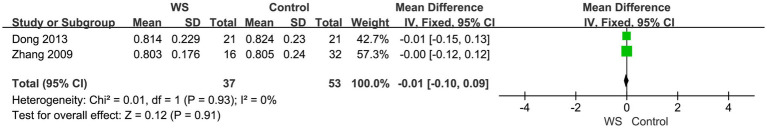
Forest map of quiet score comparison between the WS group and the control group.

Two studies, involving a total of 90 cases (37 cases in the WS group and 53 cases in the control group), compared the quiet environment scores of the WS group and the control group after CI. The fixed-effect model was used for analysis, and the combined-effect scale test result yielded *Z* = 0.12, *p* = 0.91. This indicates that there is no statistical difference between the WS group and the control group in terms of quiet environment scores, as shown in Figure 5.

#### 3.5.3 Comparison of noise environment scores

**Figure 6:**
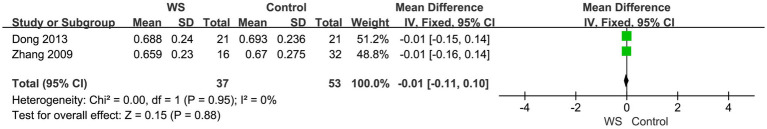
Forest map of noise score comparison between the WS group and the control group.

Two studies, involving a total of 90 cases (37 cases in the WS group and 53 cases in the control group), compared the noise environment scores of the WS group and the control group after CI. The fixed-effect model was used for analysis, and the combined-effect test result yielded *Z* = 0.15, *p* = 0.88. This suggests that there is no statistical difference between the WS group and the control group in terms of noise environment scores, as shown in Figure 6.

### 3.6 Publication bias analysis

The literature exhibits inconsistency in the included evaluation indicators. A funnel plot was generated for bias analysis of each study, and the results showed that the literature was symmetrically distributed on both sides, indicating an absence of publication bias. However, during SIR analysis, one literature source exhibited high heterogeneity.

## 4 Discussion

Waardenburg syndrome can be categorized into four types based on its clinical manifestations. Type 1 presents with ectopic inner canthus, sensorineural deafness, heterochromatic iris, white frontal hair, hypopigmentation, and straight eyebrows. Type 2 lacks ectopic inner canthus but is otherwise similar to Type 1. Type 3, also known as Klein Waardenburg syndrome, is characterized by the features of Type 1 along with muscular dysplasia and upper limb contracture, Type 4, identified as Waardenburg Shah syndrome or Waardenburg Hirschsprung disease, corresponds to Type 2 and is accompanied by Hirschsprung disease. Types 1 and 2 collectively constitute a significant proportion (16, 17). About 60% of individuals with WS types I and III suffer from sensorineural hearing loss, while 90% of those with types II and IV experience sensorineural hearing loss (18).

Some genes are believed to be related to the onset of WS. According to current research, *PAX3* is related to the pathogenesis of WS1 and WS3 (19, 20), *MITF* and *SNAI2* play a role in the pathogenesis of WS2 (21), *SOX10* is related to the pathogenesis of WS2 and WS4 (22, 23), *EDNRB* and *EDN3* are related to the pathogenesis of WS4 (24, 25). Some WS patients are accompanied by semicircular canal dysplasia, cochlear dysplasia, and large vestibular aqueduct. Structural malformations of the cochlear and labyrinth have not been reported (17). At present, most studies show that patients with WS recover well after CI, but some studies still report that the postoperative effect on patients is not good (7). Lovett et al. (2) compared the hearing and speech outcomes of WS patients before and after CI. The results showed that CI can be an effective way for improving the hearing and speech ability of WS patients. But we still want to find out whether WS and non-WS have a similar prognosis.

Both CAP and SIR were proposed by Nikolopoulos et al. of Nottingham University and filled in by patients’ relatives (26, 27). These assessments provide straightforward information about children’s hearing levels and speech abilities following surgery, making them widely used for evaluating the postoperative rehabilitation outcomes of cochlear implants (7, 8). In this study, six sets of investigations utilized CAP and SIR scores to assess auditory and speech abilities in the two groups. The results indicated no significant difference in postoperative CAP and SIR scores between the WS group and the control group.

Parents’ evaluation of aural/oral performance of children was developed by the National Acoustic Laboratory (NAL) (28) and is used for the evaluation of the auditory speech effect after cochlear implantation. Trained professionals administer the evaluation by prompting parents with questions, and responses involve describing specific cases, offering evidence of auditory speech recovery. This approach helps avoid the potential bias of direct “yes” or “no” responses, contributing to a more objective assessment (29). In this analysis, two studies utilized the PEACH score, and the results indicated no significant differences in postoperative telephone scores, quiet environment scores, and noise environment scores.

## 5 Limitations

This study has some limitations. First, the analysis is constrained by the inclusion of only a small number of studies. Second, the absence of detailed information regarding WS types and underlying genotypes hinders further discussion and exploration. Third, some of the included research samples have small populations, potentially impacting the generalizability of the findings. Fourth, in some studies, the operation time of patients was not consistently reported, introducing variability in the data. Finally, the majority of studies are from China, suggesting a geographical bias. The study calls for more diverse data from other countries to enhance the breadth and applicability of the findings.

## 6 Conclusion

There was no obvious difference in the auditory and speech recovery effects between WS patients after cochlear implantation and individuals undergoing other cochlear implant procedures. Cochlear implantation emerges as an effective method for auditory and speech therapy in WS patients, demonstrating favorable postoperative recovery effects. However, substantiating this conclusion requires a large number of high-quality disease control studies to provide robust evidence and validation.

## Data availability statement

The original contributions presented in the study are included in the article/supplementary material, further inquiries can be directed to the corresponding author.

## Author contributions

FQ: Writing – review & editing, Writing – original draft. SG: Writing – original draft. XY: Writing – original draft. XL: Writing – review & editing. JM: Writing – review & editing.

## References

[1] Waardenburg PJ. A new syndrome combining developmental anomalies of the eyelids, eyebrows and nose root with pigmentary defects of the iris and head hair and with congenital deafness. Am J Hum Genet. (1951) 3:195–253. PMID: 14902764 PMC1716407

[2] Lovett A Eastwood M Metcalfe C Muzaffar J Monksfield P Bance M. Outcomes of cochlear implantation in early-deafened patients with Waardenburg syndrome: a systematic review and narrative synthesis. Laryngoscope Investig Otolaryngol. (2023) 8:1094–107. doi: 10.1002/lio2.1110, PMID: 37621295 PMC10446317

[3] Vandamme N Berx G. From neural crest cells to melanocytes: cellular plasticity during development and beyond. Cell Mol Life Sci. (2019) 76:1919–34. doi: 10.1007/s00018-019-03049-w, PMID: 30830237 PMC11105195

[4] Eckhard A Gleiser C Rask-Andersen H Arnold H Liu W Mack A . Co-localisation of K(ir)4.1 and AQP4 in rat and human cochleae reveals a gap in water channel expression at the transduction sites of endocochlear K(+) recycling routes. Cell Tissue Res. (2012) 350:27–43. doi: 10.1007/s00441-012-1456-y, PMID: 22802001

[5] Roudbari F Dallal Amandi AR Bonyadi M Sadeghi L Jabbarpour N. SOX10Identification of a de novo, Novel Pathogenic Variant in the Splice Region of the Gene in an Iranian Azeri Turkish Family with Waardenburg Syndrome. Mol Syndromol. (2023) 14:516–22. doi: 10.1159/000531566, PMID: 38058752 PMC10697760

[6] Liberati A Altman DG Tetzlaff J Mulrow C Gøtzsche PC Ioannidis JP . The PRISMA statement for reporting systematic reviews and meta-analyses of studies that evaluate health care interventions: explanation and elaboration. PLoS Med. (2009) 6:e1000100. doi: 10.1371/journal.pmed.1000100, PMID: 19621070 PMC2707010

[7] Amirsalari S Ajallouyean M Saburi A Haddadi Fard A Abed M Ghazavi Y. Cochlear implantation outcomes in children with Waardenburg syndrome. Eur Arch Otorrinolaringol. (2012) 269:2179–83. doi: 10.1007/s00405-011-1877-3, PMID: 22159916

[8] de Sousa Andrade SM Monteiro ART Martins JHF Alves MC Santos Silva LF Quadros JMC . Cochlear implant rehabilitation outcomes in Waardenburg syndrome children. Int J Pediatr Otorhinolaryngol. (2012) 76:1375–8. doi: 10.1016/j.ijporl.2012.06.010, PMID: 22784507

[9] El Bakkouri W Loundon N Thierry B Nevoux J Marlin S Rouillon I . Cochlear implantation and congenital deafness: perceptive and lexical results in 2 genetically pediatric identified population. Otol Neurotol. (2012) 33:539–44. doi: 10.1097/MAO.0b013e31824bae35 22569142

[10] Chu YH Zhang ZF Chen CL Lan HX. Therapeutic effect of cochlear implantation in children with Waardenburg syndrome. Henan J Surg. (2017) 23:5–6. doi: 10.16193/j.cnki.hnwk.2017.04.003

[11] Dong SQ Li JN Jiao QS Sun L Liu RY Hao QQ . The effectiveness analysis of the cochlear implantation in patients with Waardenburg syndrome. Chin Sci J Hear Speech Rehab. (2013) 5:350–3. doi: 10.3969/j.issn.1672-4933.2013.05.006

[12] Gao FF Wang QR Yu SD Gu LT Shi YH Geng B. Clinical analysis of language rehabilitation in 295 cases of cochlear implantation. J Otolaryngol Ophthalmol Shandong Univ. (2018) 32:62–5.

[13] van Nierop JWI Snabel RR Langereis M Pennings RJE Admiraal RJC Mylanus EAM . Paediatric cochlear implantation in patients with Waardenburg syndrome. Audiol Neurotol. (2016) 21:187–94. doi: 10.1159/000444120, PMID: 27245679 PMC5296886

[14] Zhang ZL Cao KL Wei CG Luan L Li H. The outcomes measures of the cochlear implantation in patients with Waardenburg syndrome. J Audiol Speech Pathol. (2009) 17:372–5.

[15] Zhang XY Lin Y Xu Z Zhang YK Ren CC Zhao ZMY . Outcomes of cochlear implantation in children with Waardenburg syndrome and influencing factors. Chin J Otol. (2022) 20:720–5.

[16] Wang G Li X Gao X Su Y Han M Gao B . Analysis of genotype-phenotype relationships in 90 Chinese probands with Waardenburg syndrome. Hum Genet. (2022) 141:839–52. doi: 10.1007/s00439-021-02301-3, PMID: 34142234

[17] Lee CY Lo MY Chen YM Lin PH Hsu CJ Chen PL . Identification of nine novel variants across PAX3, SOX10, EDNRB, and MITF genes in Waardenburg syndrome with next-generation sequencing. Mol Genet Genomic Med. (2022) 10:e2082. doi: 10.1002/mgg3.2082, PMID: 36331148 PMC9747560

[18] Pingault V Ente D Dastot-Le Moal F Goossens M Marlin S Bondurand N. Review and update of mutations causing Waardenburg syndrome. Hum Mutat. (2010) 31:391–406. doi: 10.1002/humu.21211, PMID: 20127975

[19] Yang SZ Hou L Qi X Wang GJ Huang SS Zhang SS . A gross deletion of the PAX3 gene in a large Chinese family with Waardenburg syndrome type I. World J Pediatr. (2023) 19:1203–7. doi: 10.1007/s12519-023-00746-2, PMID: 37704892 PMC10590283

[20] Huang S Song J He C Cai X Yuan K Mei L . Genetic insights, disease mechanisms, and biological therapeutics for Waardenburg syndrome. Gene Ther. (2022) 29:479–97. doi: 10.1038/s41434-021-00240-2, PMID: 33633356

[21] Wen J Song J Chen J Feng Z Jing Q Gong W . Modeling of pigmentation disorders associated with MITF mutation in Waardenburg syndrome revealed an impaired melanogenesis pathway in iPS-derived melanocytes. Pigm Cell Melanoma Res. (2023) 37:21–35. doi: 10.1111/pcmr.13118, PMID: 37559350

[22] Wang Y Chai Y Zhang P Zang W. A novel variant of the SOX10 gene associated with Waardenburg syndrome type IV. BMC Med Genet. (2023) 16:147. doi: 10.1186/s12920-023-01572-1, PMID: 37365589 PMC10294417

[23] Guo M Li Q Jiang C Li S Ruan B. A de novo mutation in SOX10 in a Chinese boy with Waardenburg syndrome type 2. J Int Adv Otol. (2023) 19:255–9. doi: 10.5152/iao.2023.22745, PMID: 37272645 PMC10331715

[24] Zhang L Wan Y Wang N. Waardenburg syndrome type 4 coexisting with open-angle glaucoma: a case report. J Med Case Rep. (2022) 16:264. doi: 10.1186/s13256-022-03460-1, PMID: 35790984 PMC9258067

[25] Chandra Mohan SLN. Case of Waardenburg Shah syndrome in a family with review of literature. J Otolaryngol. (2018) 13:105–10. doi: 10.1016/j.joto.2018.05.005, PMID: 30559775 PMC6291636

[26] Archbold S Lutman ME Nikolopoulos T. Categories of auditory performance: inter-user reliability. Br J Audiol. (1998) 32:7–12. doi: 10.3109/03005364000000045, PMID: 9643302

[27] Allen MC Nikolopoulos TP O’Donoghue GM. Speech intelligibility in children after cochlear implantation. Am J Otolaryngol. (1998) 19:742–6. PMID: 9831147

[28] Ching TY Hill M. The Parents’ Evaluation of Aural/Oral Performance of Children (PEACH) scale: normative data. J Am Acad Audiol. (2007) 18:220–35. doi: 10.3766/jaaa.18.3.4, PMID: 17479615

[29] Sharma S Solanki B Solanki Y Kaurani Y. Cochlear implants: evaluation of effects of various parameters on outcomes in pediatric patients at a tertiary care centre for unilateral ear implantation. Indian J Otolaryngol Head Neck Surg. (2022) 74:360–7. doi: 10.1007/s12070-020-02129-9, PMID: 36032881 PMC9411418

